# Editorial: Current progress in genomic and genetic research on human viral diseases

**DOI:** 10.3389/fgene.2024.1407559

**Published:** 2024-04-15

**Authors:** Hezhao Ji, Birte Möhlendick, Xiang Gao, Binhua Liang

**Affiliations:** ^1^ National Microbiology Laboratory at JC Wilt Infectious Diseases Research Centre, Public Health Agency of Canada, Winnipeg, MB, Canada; ^2^ Department of Medical Microbiology and Infectious Diseases, Max Rady College of Medicine, University of Manitoba, Winnipeg, MB, Canada; ^3^ Institute of Pharmacogenetics, Essen University Hospital, Essen, Germany; ^4^ Department of Medicine, Stritch School of Medicine, Loyola University Chicago, Maywood, IL, United States; ^5^ Medical and Scientific Affairs, National Microbiology Laboratory Branch, Public Health Agency of Canada, Winnipeg, MB, Canada; ^6^ Department of Biochemistry and Medical Genetics, Max Rady College of Medicine, University of Manitoba, Winnipeg, MB, Canada

**Keywords:** genomics, genetics, human viral diseases, immunogenetics, viral evolution

Viral diseases pose a global public health threat, demanding a deeper understanding of the intricate interplay between viruses and their human hosts. The recent COVID-19 pandemic underscores the persistent challenges in combating viral pathogens and highlights the limited comprehension of their complex interactions. Insightful genomic and genetic studies are crucial in unraveling the co-evolution of viruses and hosts during infections. Such studies either delve into human genetics to investigate determinants of immune responses against viruses or explore viral genetic diversity that contributes to viral fitness, virulence, immune evasion, and viral evolution. The integration of both host and virus perspectives enhances our comprehension of the human-virus interplay, paving the way for improved preventative and therapeutic strategies. Recent advances in next-generation sequencing, bioinformatics, and data science have significantly propelled genetic and genomic studies, marking a promising era in our pursuit of combating viral infections.

This Research Topic invited a wide range of contributions, including original research, reviews, and perspectives, focusing on genetics and genomics in the context of human viral diseases. By emphasizing different aspects, the Research Topic encouraged submissions exploring evolutionary genetics to address viral evolution, genome analysis, and comparative genomics of human viruses. Key areas of interest encompassed genetic determinants influencing human susceptibility to viral infections, the impact of host genetics on vaccination response, and the exploration of viral genetics concerning infectivity, tropism, immune evasion, and antiviral resistance. Additionally, the Research Topic also welcomed new methodologies addressing viral genetic diversity and its role in viral pathogenesis. By promoting a comprehensive understanding of these genetic and genomic aspects, this Research Topic aimed to advance our knowledge and contribute to the development of effective strategies against human viral diseases.

We are delighted to have received many excellent submissions, nine of which have been successfully published in this Research Topic. This editorial piece may serve as a brief overview of these articles, highlighting their substantial contributions to the Research Topic.

This compilation includes three original research articles addressing the pressing Research Topic of COVID-19 and SARS-CoV-2. Čiučiulkaitė, et al. focused on the effect of the human *GNB3* c.825C>T polymorphism on immune responses upon COVID-19 mRNA vaccination. This was one of the few studies at the time to document the significant decline in antibody titers and T-cell responses from months 1–6 after the second dose of vaccination. It also was one of the few studies to examine the kinetics of T-cell responses against SARS-CoV-2 after mRNA vaccination. While the *GNB3* c.825C>T polymorphism had no significant effect on the humoral immune response, individuals with the CC genotype at this locus exhibited significantly enhanced T-cell responses after mRNA-1273 vaccination, suggesting improved protection against COVID-19. Möhlendick, et al. from the same research group explored the potential impact of the *GNB3* c.825C>T polymorphism on the clinical outcome of COVID-19. This study, which included 1,570 individuals, examined the potential associations between demographic factors, pre-existing conditions, laboratory parameters, *GNB3* rs5443 genotype, and COVID-19 clinical outcome. Notably, in addition to identifying associating factors against fatal COVID-19 outcomes among other examined parameters, they reported that the *GNB3* rs5443 TT genotype was significantly associated with a 40% reduced risk of COVID-19 fatality, suggesting its potential as a prognostic biomarker. While the findings of Möhlendick et al. on *GNB3* genotypes may seem to contradict the observations of Čiučiulkaitė, et al., it is important to note that the former focused on clinical outcomes in unvaccinated individuals, while the latter focuses on immune responses to COVID-19 vaccination. These divergent findings underscore the complexity of the interaction between SARS-CoV-2 and the host genetics and immune responses, and further research is needed to reconcile and fully understand these relationships.

Another COVID-19-related study was conducted by Li et al. **,** who investigated into the SARS-CoV-2 pathogenesis from a viral genetics perspective. Among many SARS-CoV-2 variants of concern (VOC), the Delta variant emerged from India in 2020 and swept the world in mid-2021 with a high transmission rate and high mortality. This study investigated the evolution of the Delta variant by analyzing mutational patterns in viral genomes originating from India at three different time periods surrounding the emergence of Delta VOC in the country. The study revealed a progressive increase in viral mutations, with viral genomes exhibiting the highest diversity following the establishment of Delta VOC. Notably, the pre-Delta phase showed a higher number of negatively selected sites, which may have protected critical gene regions during evolution. This study suggests ongoing viral adaptation and evolution and highlights the dynamic genetic landscape of SARS-CoV-2.

Not surprisingly, HIV-1 is another virus heavily studied in this Research Topic of publications. Yang et al. identified and characterized two novel unique recombinant forms (URFs) of HIV-1 from Hebei, China. Based on the analysis of near full-length genome (NFLG) sequences, both URFs were shown to result from the recombination of CRF01_AE and CRF07_BC, but with distinct recombination breakpoints. Similarly, Zhang et al. reported two novel HIV-1 URFs resulting from HIV-1 CRF01_AE and subtype B recombination in two MSM patients. Xing et al. focused their study on the origin and spread of another novel CRF - CRF68_01B initially discovered among men who have sex with men (MSM) populations. Phylodynamic analysis of CRF68_01B helped to trace the origin of CRF68_01B back to Shenzhen in 2003, indicating subsequent spread to other regions. Molecular network analysis further revealed interprovincial transmission and highlighted the significant role of MSM populations in the spread of CRF68_01B. Fan et al. conducted a study investigating integrase strand transfer inhibitor (INSTI) resistance mutations in a treatment-naive HIV-positive patient cohort in Hebei, China. This study, pertinent to the global use of INSTI-containing antiretroviral therapy, identified both major and accessory INSTI-resistance mutations, with an overall INSTI resistance rate of 3.82%. Although the frequency of INSTI resistance was low, this study highlights the need for pre-treatment testing and increased resistance monitoring during INSTI-based ART regimens. Overall, these studies have provided valuable insights into the genomic diversity, transmission dynamics, and drug resistance profiles of HIV-1, highlighting the importance of ongoing research in understanding and managing this complex viral infection.

This Research Topic includes two review articles addressing the ongoing evolution of SARS-CoV-2 and the application of quantitative metagenomics next-generation sequencing (Q-mNGS) in the detection of infectious agents causing fever of unknown origin (FUO). The review by Fang et al. outlines the trajectory evolution of SARS-CoV-2, focusing on the amino acid variations of the spike protein and genomic recombination. Pre-Omicron variants showed concentrated spike protein mutations, including early D614G, which alters the antigenicity, transmissibility, and pathogenicity of SARS-CoV-2, while Omicron introduced numerous novel mutations, enhancing transmissibility and immune evasion without increasing clinical severity. The recombinant XBB variant, emerged in 2022, likely resulted from co-circulation and co-infection in immunocompromised patients. Despite transmission advantages, these variants have demonstrated moderate antibody escape, necessitating increased surveillance for genomic variation, particularly spike protein mutations and recombination, broad-spectrum therapeutics, and widespread vaccination efforts. Dong et al. reviewed the application of Q-mNGS as a transformative method for the detection of infectious agents causing FUO. This high-throughput sequencing technology outperforms conventional molecular diagnostic methods, providing faster and more comprehensive results at a lower cost, potentially revolutionizing FUO evaluation and reducing unnecessary testing.

Collectively, we are convinced that the compilation of publications within this Research Topic provides a glimpse into the current focus and research progress in genomic and genetic studies of human viral diseases.

